# Towards biomarker-driven cancer immunotherapy: the rising of a protein phosphatase regulator

**DOI:** 10.1038/s41392-025-02425-2

**Published:** 2025-10-14

**Authors:** Xiaoyu Liu, Weiqin Yang, Alfred Sze-Lok Cheng

**Affiliations:** 1 Chongqing Key Laboratory for the Mechanism and Intervention of Cancer Metastasis, Chongqing University Cancer Hospital, Chongqing University, Chongqing, China; 2 School of Biomedical Sciences, The Chinese University of Hong Kong, Hong Kong SAR, China

**Keywords:** Tumour immunology, Tumour immunology

A recent study published in *Nature* by Dai et al. demonstrated that *PPP2R1A* mutations confer improved survival in patients with ovarian clear cell carcinoma (OCCC) treated with immune checkpoint blockade (ICB) therapy.^1^ Using serial tumor biopsies from a prospective trial of anti-PD-1 and anti-CTLA4 in OCCC patients, the authors identified inactivating somatic mutations in the protein phosphatase 2A scaffolding subunit that might predict the dual ICB response and enhance anti-tumor immunity, thus representing a new functional biomarker for cancer immunotherapy.

Identifying biomarkers for therapeutic response and resistance has been the holy grail of ICB therapy. Ovarian cancer ranks the third for incidence and the second for mortality among gynecological malignancies worldwide. Immunotherapy opened new avenues for solid cancer treatment, but a grand challenge remains for recurrent ovarian cancers, with the OCCC histological subtype having poor prognosis even when diagnosed at an early stage. Nevertheless, ‘exceptional responder patients’ with platinum-resistant OCCC who were treated with combination ICB exist,^1^ highlighting an unmet clinical need to identify the tumor-intrinsic factors that inherently determine ICB response for precision immunotherapy. Somatic mutations have been frequently detected in ovarian cancers, including *ARID1A*, *PIK3CA* and also *PPP2R1A*, which has recently been reported to sensitize OCCC to ATR inhibitor.^2^ However, the significance of *PPP2R1A* mutations in cancer immunotherapy remained unclear.

In this context, Dai and colleagues analyzed the OCCC tumor biopsies of the dual ICB trial in platinum-resistant setting (NCT03026062) and observed better overall survival in patients with *PPP2R1A* loss-of-function mutations. Transcriptional profiling of paired pre- and on-treatment tumor samples revealed enrichment of IFN-γ response signaling in *PPP2R1A*-mutated tumors at the baseline. Of note, ICB treatment further enhanced the antitumor immune responses with activation of inflammatory, complement, and allograft rejection pathways, as well as IFN-α response and IL2-STAT5 signaling. Multiplexed spatial phenotyping revealed increased infiltration of MHC-II^+^ immune cells, B cells, NK cells and the presence of tertiary lymphoid structures (TLS) in *PPP2R1A*-mutant tumors at pre-treatment stage, and an abundance of CD45RO^+^PD-1^-^CD8^+^ memory T cells particularly around MHC-I^+^ tumor cells upon ICB treatment. Consistently, lymphocytes from the long-term survivors expressed more MHC-II molecule *CD74*, co-stimulatory molecule *CD86* and functional marker *GZMB* than those from short-term survivors. Using PDX and syngeneic immunocompetent mouse models, the authors meticulously showed that either knockdown or CRISPR-edited mutation of *PPP2R1A* in tumor cells enhanced ICB and CAR-T efficacy. Finally, pan-cancer analysis corroborated that *PPP2R1A* mutations could confer prognostic values in ICB-treated patients independent of concurrent mutations (Fig. 1).

**Fig. 1:**
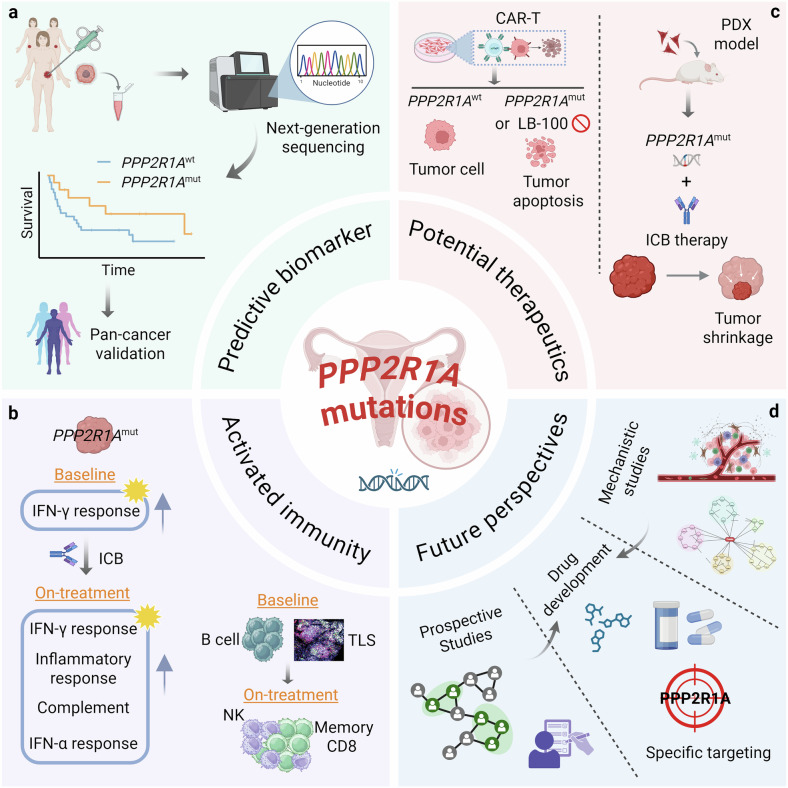
The rising of PPP2R1A in cancer immunotherapy. **a** Multi-omics analysis identified that *PPP2R1A* mutations were associated with therapeutic response and survival benefit in ICB-treated patients with ovarian cancer. **b** *PPP2R1A*-mutant tumors posed reinforced anti-tumor immunity compared with *PPP2R1A*-WT tumors as demonstrated by activated IFN-γ response signaling, enriched B cells, and TLS at the baseline and increased memory CD8^+^ T cells and NK cells after ICB therapy. **c** *PPP2R1A* mutation or inhibition sensitized tumors to CAR-T therapy in vitro and ICB therapy in PDX model in vivo. **d** Future investigations are warranted to decipher the prognostic value of *PPP2R1A* mutational status in larger prospective studies, develop selective PPP2R1A inhibitors and delineate mechanisms underlying immunotherapeutic response

This clinically-oriented study provides compelling evidence of the critical role of *PPP2R1A* mutational status in determining cancer immunotherapy outcomes of OCCC. It is worth mentioning that *PPP2R1A* is frequently mutated in other gynecological malignancies such as serous endometrial carcinomas and uterine carcinosarcoma (13–43%). Whether *PPP2R1A* mutation may serve as a biomarker of response to ICB in these tumors remains to be elucidated. Despite functional significance in ICB sensitivity, as clearly illustrated in multiple preclinical models, the exact mechanisms through which these somatic genetic changes convert the tumor microenvironment into an inflammatory niche remain unclear. Previous studies have shown that inhibition of PP2A can increase neoantigen expression on tumor cells, activate the cGAS/STING pathway, suppress regulatory T cells, and increase cytotoxic T cell activation, leading to synergistic anti-tumor effects with immunotherapy in preclinical models.^3^ However, how does PPP2R1A loss-of-function promote TLS formation with B/NK cell aggregates and increase intratumoral memory CD8^+^ T cells upon ICB therapy? *PPP2R1A* encodes the most common scaffold component of the protein phosphatase 2A (PP2A) complex, together with protein phosphatase 1 (PP1), responsible for the bulk of serine/threonine dephosphorylation in eukaryotic cells. Genomic screening uncovered IFN-γ pathway defects in tumors refractory to ICB. Future studies are thus warranted to investigate the molecular linkage between *PPP2R1A* mutations and IFN-γ signaling in the context of ICB therapy. While the elegant in vitro studies attributed the therapeutic modulation of *PPP2R1A* mutations mostly to the direct effects on tumor cells, the role of immune cell compartments cannot be excluded. Recently, another protein phosphatase component for PP1, PPP1R15A, was identified to promote ICB resistance in hepatocellular carcinoma (HCC).^4^ Interestingly, PPP1R15A was over-expressed in myeloid-derived suppressor cells. Results from the preclinical models showed that specific inhibition of PPP1R15A could break the immunosuppressive barrier to restrict HCC growth and enhance the efficacy of ICB. PPP1R15A may also function as a prognostic and predictive biomarker in patients with HCC. These studies suggest an emerging functional link between Ser/Thr protein phosphatases and cancer immunity, but the cancer-specific context such as cell types and substrates, may determine the pro- or anti-tumor effects.

This work suggests that therapeutic targeting of PPP2R1A may represent an effective strategy to improve patient outcomes after ICB or other forms of immunotherapy. The authors applied LB-100, a pharmacological inhibitor of PP2A, in in-vitro model and achieved a similar effect compared to *PPP2R1A* inhibition by knockdown or mutation. However, LB-100 primarily inhibits the catalytic subunit PP2AC, which does not fully reflect the therapeutic effect of *PPP2R1A* mutations in cancer immunity. Somatic *PPP2R1A* mutations in cancer are typically monoallelic and exert dominant-negative effects, disrupting a specific subset of PP2A holoenzymes while potentially permitting gain-of-function activities in other mutations.^5^ However, complete knockout of both *PPP2R1A* alleles is non-viable. While LB-100 is a valuable tool to phenocopy the *PPP2R1A* mutations, development of selective PPP2R1A inhibitors would be an attractive avenue of cancer drug discovery for *PPP2R1A*-WT gynecological cancer patients. On the other hand, similar to *EGFR* mutations for targeted therapy using tyrosine kinase inhibitors, patients with loss-of-function *PPP2R1A* mutations may give rise to the sought-after biomarker for immunotherapy.

## References

[1] Dai, Y. et al. PPP2R1A mutations portend improved survival after cancer immunotherapy. *Nature* **644**, 537–546 (2025). 40604275 10.1038/s41586-025-09203-8 PMC12350166

[2] Stewart, J. et al. PPP2R1A mutations cause ATR inhibitor sensitivity in ovarian clear cell carcinoma. *Oncogene* **44**, 618–629 (2025). 39939726 10.1038/s41388-024-03265-0 PMC11850283

[3] Clark, M. C. et al. A combination of protein phosphatase 2A inhibition and checkpoint immunotherapy: a perfect storm. *Mol. Oncol.* **18**, 2333–2337 (2024). 38932511 10.1002/1878-0261.13687 PMC11459031

[4] Liu, X. et al. PPP1R15A-expressing monocytic MDSCs promote immunosuppressive liver microenvironment in fibrosis-associated hepatocellular carcinoma. *JHEP Rep.* **6**, 101087 (2024). 38882672 10.1016/j.jhepr.2024.101087 PMC11179254

[5] Haesen, D. et al. Recurrent mutations in uterine cancer act through a dominant-negative mechanism to promote malignant cell growth. *Cancer Res.* **76**, 5719–5731 (2016). 27485451 10.1158/0008-5472.CAN-15-3342

